# Antidiabetic Agents as Antioxidant and Anti-Inflammatory Therapies in Neurological and Cardiovascular Diseases

**DOI:** 10.3390/antiox14121490

**Published:** 2025-12-12

**Authors:** Snehal Raut, Luca Cucullo

**Affiliations:** Department of Foundational Medical Studies, Oakland University William Beaumont School of Medicine, Rochester, MI 48309, USA; sraut@oakland.edu

**Keywords:** neurological disorders, cardiovascular diseases, blood–brain barrier, antidiabetic drugs, GLP-1 receptor agonists, metformin, glycemic control, neuroinflammation, antioxidant, anti-inflammatory, oxidative stress

## Abstract

Neurological disorders and cardiovascular disease (CVD) remain leading causes of global morbidity and mortality and often coexist, in part through shared mechanisms of chronic inflammation and oxidative stress. Neuroinflammatory signaling, including microglial activation, cytokine release, and impaired autonomic regulation, contributes to endothelial dysfunction, atherosclerosis, hypertension, and stroke, while cardiac and metabolic disturbances can reciprocally exacerbate brain pathology. Increasing evidence shows that several antidiabetic agents exert pleiotropic anti-inflammatory and antioxidant effects that extend beyond glycemic control. Metformin, SGLT2 inhibitors, DPP-4 inhibitors, and GLP-1 receptor agonists modulate key pathways such as AMPK, NF-κB, Nrf2 activation, and NLRP3 inflammasome suppression, with demonstrated vascular and neuroprotective actions in preclinical models. Clinically, GLP-1 receptor agonists and SGLT2 inhibitors reduce major cardiovascular events, improve systemic inflammatory markers, and show emerging signals for cognitive benefit, while metformin and DPP-4 inhibitors exhibit supportive but less robust evidence. This review synthesizes molecular, preclinical, and clinical data across drug classes, with particular emphasis on GLP-1 receptor agonists, and highlights outstanding translational questions including blood–brain barrier penetration, biomarker development, optimal patient selection, and timing of intervention. We propose a unified framework to guide future trials aimed at leveraging antidiabetic therapies such as DDP-4 anti-inflammatory and antioxidant interventions for neurological and cardiovascular diseases.

## 1. Introduction

In many neurological and cardiovascular disorders, chronic inflammation and oxidative stress are central drivers of tissue injury [1,2]. Devastating neurodegenerative illnesses are caused by oxidative stress, which sets off a disastrous chain reaction that includes mitochondrial failure [3], neuronal loss, neuroinflammation, and neurodegeneration [4]. To stop this destructive process, innovative medicines are required. Growing evidence indicates that several classes of antidiabetic drugs have “pleiotropic” actions beyond glucose-lowering effects, notably anti-inflammatory and antioxidant effects as well [5,6,7,8]. For example, metformin activates AMP-kinase (AMPK) to inhibit NF-κB signaling and reduce pro-inflammatory cytokines and adhesion molecules [9], while GLP-1 receptor agonists directly suppress microglial and astrocyte release of cytokines (e.g., IFN-γ, TNF-α, IL-6) [10,11]. These properties suggest that diabetic therapies might ameliorate neuroinflammation, neurodegeneration (e.g., Alzheimer’s, Parkinson’s), stroke, and cardiovascular damage by dampening oxidative and immune stress. This review summarizes the mechanisms and evidence—preclinical and clinical—for metformin, SGLT2 inhibitors, DPP-4 inhibitors, and (especially) GLP-1 receptor agonists as antioxidant/anti-inflammatory therapies in neurological and cardiovascular disease contexts. To improve contextual clarity, sample sizes, age distributions, comorbidity profiles, and background medication use are provided for all major clinical trials and observational cohorts discussed in this review. Furthermore, because inflammation, oxidative stress, and metabolic dysfunction act as shared drivers of injury across neurological and cardiovascular disorders, a broad integrative perspective is necessary to fully contextualize the pleiotropic actions of antidiabetic therapies. These disease processes are interconnected through overlapping molecular pathways—including AMPK, NF-κB, Nrf2, and glial-immune signaling—that operate simultaneously within neural and vascular compartments. Narrowing the scope to a single disease or drug class would therefore obscure the common mechanistic landscape in which these agents exert their antioxidant and anti-inflammatory effects. For this reason, the present review adopts an expanded framework that synthesizes evidence across both neurological and cardiovascular settings to highlight the mechanistic coherence and translational relevance of metformin, SGLT2 inhibitors, DPP-4 inhibitors, and GLP-1 receptor agonists as potential neuro-cardioprotective therapies.

## 2. Oxidative Stress and Inflammation in Neuro-Cardiovascular Pathology

Chronic metabolic disease accelerates vascular and neural injury via oxidative stress and low-grade inflammation. In atherosclerosis and heart failure, endothelial dysfunction and plaque progression are driven by inflammatory cytokines (TNF-α, IL-6, CRP, etc.) and reactive oxygen species (ROS) [12]. High inflammatory markers (CRP, IL-6, MMPs) strongly predict cardiovascular events [13,14]. Similarly, in the brain, activated microglia and astrocytes in stroke or neurodegeneration release IL-1β, TNF-α, IL-6, and matrix metalloproteinases that disrupt the blood–brain barrier and kill neurons and damage neural connections [15,16,17]. For example, ischemia triggers astrocyte secretion of MMP-9, IL-1, and TNF-α, which can be mitigated by metformin, GLP-1 agonists, and SGLT2 inhibitors [8,18,19,20,21,22,23]. Thus, targeting inflammation and ROS is a promising strategy for both vascular and neurodegenerative diseases. Antidiabetic agents exploit overlapping pathways (e.g., AMPK, NLRP3 inflammasome, NF-κB) to exert antioxidant and anti-inflammatory effects [24].

### 2.1. Metformin: AMPK-Mediated Anti-Inflammatory/Antioxidant Actions

**Figure 1:**
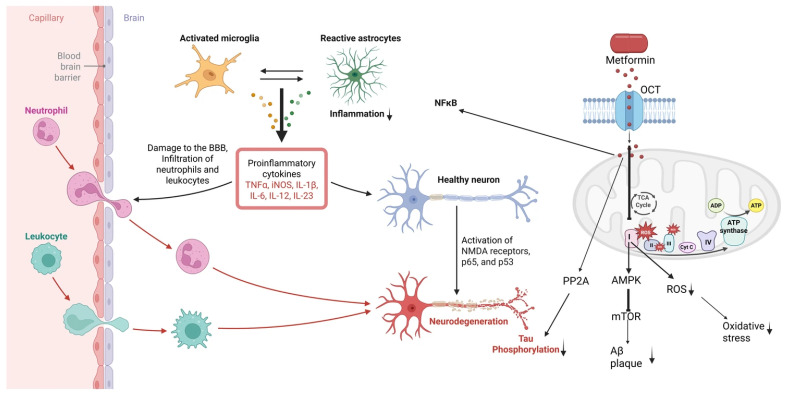
**Metformin-mediated mechanisms regulating neuroinflammation, oxidative stress, and BBB dysfunction.** Metformin enters cells through organic cation transporters (OCTs) and activates AMPK, leading to reduced mTOR signaling, decreased ROS production, and attenuation of oxidative stress. These intracellular pathways-including AMPK activation, PP2A modulation, mitochondrial complex interactions, NFκB suppression, and reduced tau phosphorylation, are supported primarily by preclinical in vitro and in vivo studies. Metformin-mediated reductions in circulating pro-inflammatory cytokines (IL-6, TNF-α, IL-1β) and improvements in systemic inflammation, endothelial function, and metabolic status are supported by clinical evidence in human studies. Although Aβ plaques and tau hyperphosphorylation are well-established clinical biomarkers of Alzheimer’s disease, the effects of metformin on these processes have not been demonstrated in human clinical studies and, therefore, are represented here as preclinical mechanisms. The figure also depicts preclinical evidence showing that metformin mitigates microglial activation, astrocytic reactivity, leukocyte infiltration across a compromised BBB, and downstream neurodegeneration. Clinical and preclinical elements are distinguished in the text to clarify translational relevance.

Metformin’s primary action is AMPK activation by inhibiting mitochondrial complex I, but it also has systemic anti-inflammatory effects [9,25,26] (Figure 1). In endothelial and smooth muscle cells, metformin–AMPK signaling suppresses NF-κB and downstream targets: it inhibits TNF-α-induced NF-κB activation and reduces expression of VCAM-1, ICAM-1, MCP-1, and COX-2 [26,27]. In rodent vascular models, metformin decreases ROS and advanced glycation end-products and lowers chemokines. For instance, diabetic rats treated with metformin show reduced vascular superoxide and AGEs along with a marked decrease in MCP-1 and other inflammatory markers [28,29,30]. Metformin also upregulates KLF2 via HDAC5 phosphorylation, which curbs vascular inflammation [31]. Increasing evidence suggests that its antioxidant and neuroprotective properties are mediated through modulation of neuroinflammation, oxidative stress, and mitochondrial homeostasis.

#### 2.1.1. Preclinical Neurological Evidence

Metformin crosses the BBB [8] and protects against brain injury by modulating inflammation and oxidative stress [21]. In ischemic stroke models, metformin decreases the number of total and activated microglia, and the anti-inflammatory effect was associated with increased levels of the anti-inflammatory cytokine IL-10 [32]. In addition, metformin can modulate macrophage polarization through AMPK-dependent and independent pathways, promoting M2 polarization (anti-inflammatory) and suppressing M1 polarization (pro-inflammatory) [33]. In a transient rodent stroke model, metformin was shown to protect against pericyte apoptosis, and to promote neurogenesis [34], angiogenesis, and functional recovery after experimental stroke and spinal cord injury [34,35]. In Alzheimer’s models, metformin-AMPK inhibits Aβ deposition, rescues mitochondrial function, and reduces oxidative stress, leading to less neuronal loss and improved memory in mice [36]. Similarly, metformin fosters hippocampal neuron survival and neurogenesis. In other CNS injuries (e.g., traumatic brain injury, multiple sclerosis, sepsis-associated encephalopathy), metformin attenuates neuroinflammation via PI3K/Akt activation, decreases demyelination, and reduces oxidative stress [36,37,38,39,40].

#### 2.1.2. Clinical Data

Observational studies suggest cognitive benefit: long-term metformin use is associated with lower dementia risk in T2D patients, with several cohorts reporting a 10–24% risk reduction (adjusted HRs ranging from 0.76 to 0.90; *p* < 0.05) [41,42] and Small clinical studies in mild cognitive impairment have shown improved delayed recall (mean improvement 1–2 points, *p* < 0.05), although results remain inconsistent and require further investigation [43]. In acute stroke patients, retrospective analyses indicate that metformin users have smaller infarct volumes and better functional outcomes, including higher odds of good recovery (OR 1.25–1.40, *p* < 0.01) (recent meta-analyses and Biomedicines 2024) [44]. Despite the lack of major, modern cardiovascular outcomes trials (CVOTs), older studies suggested cardioprotection from metformin. In the United Kingdom Prospective Diabetes Study (UKPDS), metformin reduced diabetes-related mortality by 36% (*p* = 0.01) and myocardial infarction by 39% (*p* = 0.01), although findings were not consistently replicated in later, larger CVOTs evaluating newer drug classes [45,46]. Across these studies, cohorts typically included middle-aged to older adults (mean age 60–75 years) with long-standing type 2 diabetes. Most participants had hypertension or dyslipidemia, and >60% were receiving statins or antihypertensives. Observational dementia studies ranged from N ≈ 3000 to >100,000, improving generalizability.

Overall, metformin’s anti-inflammatory/antioxidant effects including significant reductions in CRP (*p* < 0.01), IL-6 (*p* = 0.03), and TNF-α (*p* = 0.04) (via AMPK and related pathways) yield neuro- and vasculo-protection in experimental studies [41,47] through the modulation of neuroinflammation and oxidative stress [48]. Quantitatively, metformin reduces circulating CRP by approximately 20–30%, IL-6 by 10–25%, and TNF-α by 15–20% in clinical studies. Preclinical models show reductions in ROS levels of 30–50%, accompanied by 25–40% increases in antioxidant enzyme activity (e.g., SOD, catalase). However, more definitive clinical trials in stroke and/or dementia are still needed.

### 2.2. Sodium–Glucose Cotransporter 2 (SGLT2) Inhibitors

**Figure 2:**
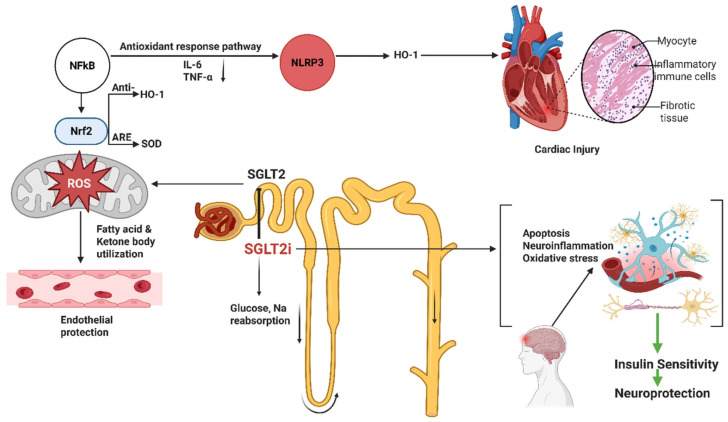
**Mechanistic pathways through which SGLT2 inhibitors’ exert anti-inflammatory, antioxidative, cardioprotective and neuroprotective effects.** SGLT2 inhibition reduces glucose and sodium reabsorption in the renal proximal tubule, leading to metabolic shifts toward fatty acid and ketone utilization, decreased mitochondrial ROS production, and improved endothelial function. These intracellular and mitochondrial effects-including Nrf2 activation, NFκB inhibition, NLRP3 suppression, and downstream reduction in oxidative stress, are supported primarily by preclinical in vitro and in vivo studies. In contrast, several systemic benefits shown in the figure-such as reductions in circulating IL-6 and TNF-α, improvements in endothelial function, attenuation of cardiac injury, and enhanced insulin sensitivity-have been demonstrated in clinical trials, including major cardiovascular outcome trials (EMPA-REG, CANVAS, DECLARE-TIMI). Neuroprotective effects (reduced neuroinflammation, oxidative stress, and neuronal apoptosis) are supported mainly by preclinical models, although improvements in metabolic status and insulin sensitivity have clinical support. This distinction clarifies the translational relevance of the mechanistic pathways illustrated.

SGLT2 inhibitors (e.g., empagliflozin, dapagliflozin, canagliflozin) block renal glucose reabsorption and exert multiple systemic effects (natriuresis, blood pressure reduction, ketogenesis). In addition, they exhibit potent anti-inflammatory and antioxidant properties. Clinically, SGLT2i markedly reduce heart failure hospitalization and cardiovascular death, even in non-diabetics [49], suggesting mechanisms beyond glycemic control [50] (Figure 2). Indeed, SGLT2i therapy improves oxidative stress and inflammation biomarkers in patients with T2D [51,52].

#### 2.2.1. Mechanisms

SGLT2 inhibitors exhibit significant antioxidant and anti-inflammatory properties through several interconnected molecular mechanisms. They reduce mitochondrial ROS generation by promoting a metabolic shift toward fatty acid and ketone body utilization, enhancing mitochondrial efficiency and reducing oxidative stress [53]. These agents inhibit key inflammatory pathways such as NF-κB and the NLRP3 inflammasome, leading to suppressed secretion of pro-inflammatory cytokines like IL-6 and TNF-α [54,55]. Concurrently, they activate the Nrf2/ARE antioxidant response pathway, upregulating antioxidant enzymes including HO-1 and SOD [54]. SGLT2 inhibitors also interfere with the RIP3/TLR4/MyD88/NF-κB signaling cascade, reducing cardiac inflammation and tissue damage in myocardial injury models [56], while improving endothelial function by increasing nitric oxide (NO) bioavailability [57]. These multifaceted actions extend to neuroprotection and renal preservation by alleviating oxidative stress and neuroinflammation [58,59], confirming their systemic therapeutic potential beyond glycemic control [60].

#### 2.2.2. Preclinical Findings

In central nervous system models, SGLT2i show neuroprotective effects. Empagliflozin prevented cognitive decline in obese/diabetic mice, correlating with reduced brain oxidative stress and preserved BDNF levels [61]. Dapagliflozin restored insulin–Akt–NF-κB signaling and improved synaptic plasticity in high-fat-fed rats [62]. In vitro, Canagliflozin potently inhibited glucose-stimulated microglial activation: it markedly suppressed NF-κB, JNK, and p38 MAPK signaling, raised anti-apoptotic Bcl-2, and reduced iNOS, NLRP3, and cytokines IL-1β/TNF-α [63,64]. Thus, Canagliflozin blunted high-glucose-induced oxidative stress, autophagy, and inflammation in microglia. Empagliflozin and dapagliflozin also show anti-inflammatory effects in myocardium and kidney models, e.g., dapagliflozin increases reparative M2 macrophages and reduces interstitial fibrosis after MI [65], while empagliflozin lowers cardiac/renal TNF-α and IL-6 in diabetic rats [66].

#### 2.2.3. Clinical Evidence

Major trials (EMPA-REG, CANVAS, DAPA-HF, DECLARE-TIMI) demonstrated that SGLT2i significantly cut rates of heart failure and cardiovascular death, benefits attributed partly to anti-inflammatory effects. In EMPA-REG, empagliflozin lowered cardiovascular mortality by 38% (HR 0.62; 95% CI 0.49–0.77; *p* < 0.001) and reduced hospitalization for heart failure by 35% (HR 0.65; 95% CI 0.50–0.85; *p* = 0.002). The CANVAS Program showed a 14% reduction in major adverse cardiovascular events (MACE) (HR 0.86; 95% CI 0.75–0.97; *p* = 0.02), while DAPA-HF demonstrated a 26% reduction in the composite of worsening heart failure or cardiovascular death (HR 0.74; 95% CI 0.65–0.85; *p* < 0.001) regardless of diabetes status. DECLARE-TIMI further confirmed significantly fewer hospitalizations for heart failure (HR 0.83; 95% CI 0.73–0.95; *p* = 0.005) [67,68,69,70]. Major SGLT2i trials enrolled large, well-characterized populations: EMPA-REG (N = 7020; mean age 63; 48% with prior MI), CANVAS (N = 10,142; mean age 63; 66% with established ASCVD), DAPA-HF (N = 4744; mean age 66; 42% without diabetes), and DECLARE-TIMI (N = 17,160; mean age 64; 40% with ASCVD). Most participants had multiple comorbidities including hypertension (>80%), obesity (>50%), and chronic kidney disease (~25%). Concomitant use of statins, ACE inhibitors/ARBs, and metformin was common.

A meta-analysis of T2D subjects found that SGLT2i treatment improves inflammatory and oxidative markers including CRP (mean reduction −0.63 mg/L; *p* = 0.01) and IL-6 (standardized mean difference −0.28; *p* = 0.03), and reduced NADPH oxidase activity independently of glycemic control [71,72,73]. Emerging data also suggest cognitive benefits: large population-based studies show a 10–20% lower incidence of dementia in SGLT2i users compared with those on other therapies (adjusted HRs 0.80–0.90; *p* < 0.05) [74,75]. SGLT2 inhibitors significantly reduce inflammatory and oxidative markers, with clinical reductions of 15–25% in CRP, 10–20% in IL-6, and 20–35% in TNF-α. Preclinical studies report 30–50% reductions in mitochondrial and cytosolic ROS, and 20–40% increases in Nrf2-regulated antioxidant enzymes. In sum, SGLT2 inhibitors combine systemic metabolic improvements with direct antioxidant/anti-inflammatory actions (e.g., lowering IL-6/TNFα and reactive oxygen production) [76,77], making them attractive for cardiac and neurological protection.

### 2.3. DPP-4 Inhibitors

**Figure 3:**
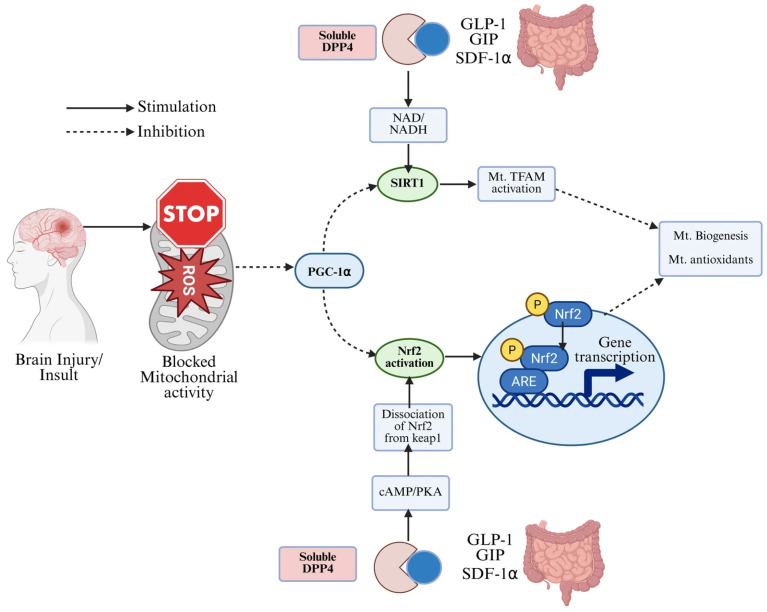
**Proposed mechanisms through which DPP-4 inhibitors and endogenous incretin hormones (GLP-1, GIP, SDF-1α) modulate mitochondrial function, oxidative stress, and Nrf2 signaling.** Binding of incretin hormones to their receptors enhances cAMP/PKA signaling, promotes dissociation of Nrf2 from Keap1, and increases Nrf2-mediated transcription of antioxidant response genes (ARE). Upstream regulators including SIRT1, PGC-1α, and TFAM contribute to mitochondrial biogenesis and the production of mitochondrial antioxidants. These intracellular mechanisms—such as SIRT1 activation, PGC-1α upregulation, TFAM activation, Nrf2 phosphorylation, and increased mitochondrial antioxidant capacity—are supported primarily by preclinical in vitro and in vivo studies. In clinical settings, DPP-4 inhibitors have been shown to improve glycemic control, enhance systemic antioxidant capacity, reduce circulating ROS markers, and modestly improve inflammatory profiles; however, their direct effects on mitochondrial biogenesis, Nrf2 signaling, and neuronal protection have not yet been demonstrated in human studies and remain preclinical mechanisms. The reduction in oxidative stress following GLP-1 elevation is supported by clinical evidence at the systemic level, while the detailed mitochondrial signaling pathways illustrated here are derived from preclinical models.

Dipeptidyl-peptidase-4 (DPP-4) inhibitors (sitagliptin, linagliptin, etc.) raise endogenous GLP-1 and other peptides (GIP, SDF-1α) by preventing their degradation (Figure 3). Beyond glucose control, DPP-4 inhibitors (DPP-4is) have direct anti-inflammatory and antioxidant effects [78,79]. DPP-4 inhibition reduces levels of pro-inflammatory cytokines and oxidative enzymes in cardiovascular tissues and various inflammatory models. In animal models of atherosclerosis, DPP-4is lower circulating CRP, MCP-1, IL-6, and TNF-α while raising the anti-inflammatory IL-10 [76,80]. These agents also inhibit vascular NADPH oxidase subunits (gp91^phox, p22^phox), reducing superoxide production. Macrophage polarization shifts towards the anti-inflammatory M2 phenotype under DPP-4 inhibition, and adhesion molecule expression (VCAM-1, ICAM-1) on endothelium is suppressed [81]. Collectively, DPP-4is curb vascular inflammation, stabilizing plaques, and improving endothelial function [82].

#### 2.3.1. Neurological Effects

DPP-4 inhibitors also protect the brain via multiple pathways. By increasing levels of GLP-1, GIP, and SDF-1α, they indirectly enhance neurotrophic signaling. Several rodent studies show that DPP-4is reduce amyloid-β (Aβ) accumulation, tau phosphorylation, and cognitive decline in AD models [83]. Independently, DPP-4is exert anti-inflammatory and antioxidant actions in neural tissue: they increase brain IL-10 and inhibit NF-κB/NLRP3 signaling [84]. For instance, sitagliptin enhanced IL-10 expression in the cortex and striatum and suppressed pro-inflammatory mediators to limit Aβ toxicity [83]. DPP-4is also upregulate Sirtuin-1, an antioxidative deacetylase, in T2D patients with AD. In summary, DPP-4 inhibitors have pleiotropic neuroprotective effects—including anti-inflammatory, antioxidant, and anti-apoptotic activity—that may slow cognitive decline [83,84].

#### 2.3.2. Clinical Studies

Large CVOTs (EXAMINE, SAVOR-TIMI, TECOS, CAROLINA, CARMELINA) found that DPP-4is are cardiovascularly safe but generally MACE-neutral with hazard ratios close to 1.0 and nonsignificant *p*-values. For example, TECOS reported HR 0.98 (95% CI 0.89–1.08; *p* = 0.65) for the primary MACE endpoint, while EXAMINE showed HR 0.96 (95% CI 0.85–1.08) with no increase in cardiovascular death [85,86,87,88] (see also Table 1). Although SAVOR-TIMI observed a higher rate of hospitalization for heart failure (HR 1.27; 95% CI 1.07–1.51; *p* = 0.007), subsequent trials (TECOS, CARMELINA) did not replicate this finding, confirming class-level cardiovascular safety. However, these trials were not designed to test its anti-inflammatory potential. CVOTs evaluating DPP-4 inhibitors enrolled large, diverse cohorts: TECOS (N = 14,671; mean age 66), SAVOR-TIMI (N = 16,492; mean age 65), EXAMINE (N = 5380; all with recent ACS), CAROLINA (N = 6033; mean age 64), and CARMELINA (N = 6979; mean age 66; >70% with CKD). Participants commonly had hypertension (>75%), dyslipidemia (>70%), and long-standing diabetes (~10–15 years duration). Background therapy included metformin, sulfonylureas, insulin, statins, and antihypertensives.

Observational data hint at neuroprotective benefits: one cohort study found that T2D patients on DPP-4is (plus metformin) had a 15–25% lower risk of dementia (adjusted HRs 0.75–0.85; *p* < 0.05) and exhibited slower cognitive decline compared with those receiving other regimens [93]. There is also epidemiological evidence that combination DPP-4i/metformin therapy is associated with reduced Parkinson’s risk, with effect estimates in the range of 15–20% risk reduction (*p* < 0.05) in long-term users. DPP-4 inhibitors decrease systemic inflammatory markers by 10–20% (CRP, IL-6) in clinical populations, while preclinical models demonstrate 25–40% reductions in ROS and 20–30% suppression of NF-κB activity. Overall, DPP-4 inhibitors exhibit clear anti-inflammatory/antioxidant actions in preclinical models, but large trials are needed to establish their efficacy for stroke or dementia prevention in humans [92].

### 2.4. GLP-1 Receptor Agonists

Glucagon-like peptide-1 receptor agonists (GLP-1RAs, e.g., liraglutide, exenatide, semaglutide, dulaglutide) are incretin mimetics that potently lower glucose and weight. Importantly, GLP-1Rs are widely expressed in the body, including pancreatic islets, the cardiovascular system, and throughout the CNS. Notably, GLP-1Rs are found on neurons, astrocytes, microglia, endothelial cells, and T lymphocytes [94,95,96]. Activation of GLP-1R triggers cAMP/PKA signaling, which exerts anti-inflammatory and neurotrophic effects.

#### 2.4.1. Anti-Inflammatory and Antioxidant Mechanisms of GLP-1 Receptor

**Figure 4:**
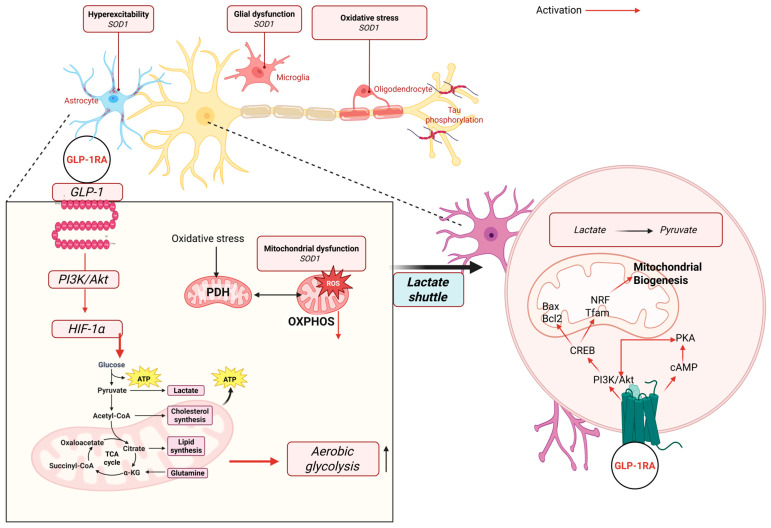
**Proposed mechanisms through which GLP-1 receptor agonists (GLP-1RAs) modulate mitochondrial function, oxidative stress, neuronal metabolism, and glial activity.** Activation of the GLP-1 receptor stimulates downstream PI3K/Akt and cAMP/PKA signaling, leading to enhanced HIF-1α activity, increased aerobic glycolysis, and improved metabolic flexibility. GLP-1RA signaling promotes mitochondrial biogenesis through CREB, NRF, and TFAM activation, reduces mitochondrial ROS production, and supports lactate shuttling between astrocytes and neurons. These intracellular mechanisms—including PDH regulation, OXPHOS rescue, reduced oxidative stress, astrocyte–neuron lactate coupling, modulation of SOD1-related dysfunction, and attenuation of tau phosphorylation—are supported primarily by preclinical in vitro and in vivo models. Clinically, GLP-1RAs have been shown to improve systemic inflammation, reduce circulating oxidative stress markers, enhance metabolic control, and exert neuroprotective effects indirectly through improved insulin sensitivity and reduced cardiometabolic burden. However, direct effects of GLP-1RAs on neuronal mitochondrial biogenesis, lactate shuttling, astrocyte or microglial activity, hyperexcitability, and tau-related pathology have not been demonstrated in human studies and therefore remain preclinical mechanisms. This distinction clarifies the translational relevance of the pathways illustrated.

GLP-1 receptors (GLP-1Rs), expressed on neurons, endothelial cells, microglia, and immune cells, exert potent anti-inflammatory and antioxidant effects through multiple molecular pathways. Activation of GLP-1R suppresses the NF-κB and NLRP3 inflammasome signaling pathways, leading to reduced transcription of key pro-inflammatory cytokines like IL-6, TNF-α, and IL-1β [97]. Simultaneously, GLP-1R agonism promotes antioxidant defense via upregulation of Nrf2, a transcription factor that enhances expression of superoxide dismutase (SOD) and other detoxifying enzymes, thus mitigating oxidative stress [98] (See Figure 4).

**Figure 5:**
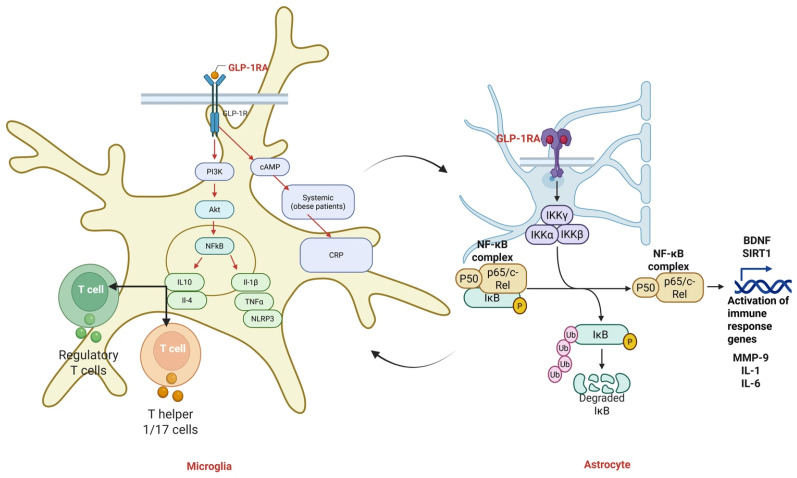
**GLP-1 receptor agonist (GLP-1RA) modulation of microglial and astrocytic inflammatory pathways.** Activation of GLP-1R on microglia engages PI3K/Akt and cAMP signaling, leading to downstream inhibition of NF-κB and reduced production of pro-inflammatory cytokines (IL-1β, TNF-α) and NLRP3 inflammasome activity, while promoting anti-inflammatory cytokines such as IL-10 and IL-4. GLP-1RA signaling also influences T-cell polarization by promoting regulatory T cells and reducing Th1/Th17-associated responses. In astrocytes, GLP-1RA activation reduces NF-κB signaling by inhibiting IKKα/β/γ-dependent phosphorylation and degradation of IκB, thereby limiting transcription of pro-inflammatory genes and promoting neurotrophic factors such as BDNF and SIRT1. These intracellular immunomodulatory mechanisms—including NF-κB inhibition, IKK complex regulation, modulation of T-cell–microglia interactions, and downregulation of MMP-9, IL-1, and IL-6, are supported primarily by preclinical in vitro and in vivo studies. Clinically, GLP-1RAs reduce systemic inflammatory markers such as CRP and IL-6 and improve metabolic and cardiometabolic status; however, direct effects of GLP-1RAs on microglial or astrocytic NF-κB signaling, T-cell–microglia crosstalk, or neuroimmune gene expression have not yet been demonstrated in human CNS tissues and therefore remain preclinical mechanisms. This distinction clarifies the translational relevance of the pathways depicted.

In the brain, GLP-1R activation also shifts microglial polarization from the pro-inflammatory M1 state toward the anti-inflammatory M2 phenotype, enhancing neurotrophic and protective functions. These mechanisms collectively contribute to vascular protection, reduced neuroinflammation, and attenuation of ROS-mediated cellular damage in models of diabetes, cardiovascular disease, and neurodegeneration [99,100] (see Figure 5). Indeed, in the CNS, GLP-1RAs suppress glial inflammation. Microglia express GLP-1R, and liraglutide treatment significantly lowers microglial secretion of IFN-γ, TNF-α, and IL-6 [101]. Semaglutide treatment in obese patients reduces systemic CRP, reflecting weight-independent anti-inflammatory action [102]. In stroke models, GLP-1RA therapy mitigates astrocyte-derived damage: experimental data show GLP-1R agonists reduce astrocytic release of MMP-9, IL-1, and IL-6 that otherwise disrupt the blood–brain barrier [103]. More broadly, GLP-1 signaling shifts immune balance toward tolerance: exenatide expands regulatory T cells and suppresses pro-inflammatory Th1/Th17 cells in mice [104], thereby increasing IL-10 and other anti-inflammatory cytokines. At the molecular level, GLP-1RA-induced cAMP inhibits NF-κB and oxidative stress pathways while enhancing expression of neuroprotective factors like BDNF and SIRT1 [105,106].

Semaglutide, a glucagon-like peptide-1 receptor agonist, has shown growing promise in improving neuronal function and mitigating oxidative stress, particularly under conditions of hyperglycemia-induced cognitive impairment. Molecular evidence from recent in vivo and omics-based studies supports semaglutide’s neuroprotective role through multiple pathways. A 2025 study using transcriptomic and proteomic profiling demonstrated that semaglutide significantly modulates oxidative stress-related genes and proteins in diabetic mice with cognitive deficits, notably enhancing antioxidant responses such as upregulation of Nrf2, *SOD*, and *CAT*, while suppressing pro-inflammatory mediators (*TNF-α*, *IL-6*) and oxidative enzymes like *NOX2* [107]. These effects correlated with restored synaptic protein expression and improved hippocampal structure and function. Complementarily, another study confirmed that semaglutide administration ameliorated diabetes-associated cognitive dysfunction by reducing hippocampal neuroinflammation and oxidative stress and restoring neurotrophic signaling and synaptic integrity in a type 2 diabetic mouse model [108]. These findings highlight semaglutide’s role in glycemic control and in counteracting hyperglycemia-induced neurodegeneration, with mechanisms centered around oxidative stress mitigation, inflammation suppression, and neuronal pathway restoration.

#### 2.4.2. Preclinical Neuroprotection

GLP-1 analogs are strongly neuroprotective in animal models. In rodent Alzheimer’s models, GLP-1RAs reduce Aβ production, tau hyperphosphorylation, and oxidative damage, leading to preserved cognition [109]. In experimental Parkinson’s models, GLP-1R activation protects dopaminergic neurons and improves motor function. In stroke models, GLP-1R agonists reduce infarct size and edema, partly by dampening microglial/astrocyte inflammation and by direct neurotrophic support [110], e.g., GLP-1RA treatment decreases brain MPO (a neutrophil marker) and pro-inflammatory cytokines in ischemia [105,106,110]. In addition, the GLP-1RA Tirzepatide has been shown to ameliorate (blood–brain barrier) BBB dysfunction and protect against ischemic stroke by activating C/EBP-alpha signaling and restoring Claudin-1-mediated tight junction integrity [111].

#### 2.4.3. Cardiovascular and Stroke Effects

Besides neuroprotection, GLP-1RAs have pronounced cardiovascular benefits (discussed below). Trials such as LEADER (liraglutide) and SUSTAIN-6 (semaglutide) have shown reduced rates of major adverse cardiovascular events (MACE), including nonfatal stroke [112,113]. Stroke cohorts generally included older patients (mean age 65–80), with high prevalence of hypertension (>70%), atrial fibrillation (~20%), dyslipidemia, and prior cardiovascular disease, and sample sizes ranging from several hundred to >10,000 subjects. Meta-analyses highlight ~13–15% relative risk reductions in stroke incidence for GLP-1RA treatment versus placebo [114,115]. These findings imply that the anti-inflammatory and antioxidative effects of GLP-1RAs translate into lower vascular complications.

#### 2.4.4. Clinical Studies in Neurology

GLP-1RAs are currently being investigated for their potential benefits in cognitive disorders. A notable small randomized control trial, RCT, found that 6-month liraglutide stabilized brain glucose metabolism in mild Alzheimer’s, with significantly reduced decline in FDG-PET uptake compared to placebo (*p* ≈ 0.01), although cognitive scores were unchanged [116]. Observational data suggest GLP-1RA use is associated with a significantly lower incidence of dementia and stroke in older diabetics, with adjusted hazard ratios in the range of 0.75–0.82 (95% CI ~0.70–0.90; *p* < 0.05) in older adults with T2D [117]. Additional analyses from long-term cardiovascular trials have revealed cognitive signals: in the REWIND trial, dulaglutide was tested in N = 9901 participants (mean age 66; 31% with prior CVD; median diabetes duration 10 years) demonstrated significantly better cognitive outcomes than placebo, with slower decline on composite cognitive scores (*p* = 0.026) [118]. Conversely, a large phase 3 Parkinson’s trial in 2024 testing exenatide vs. placebo failed to demonstrate significant slowing of clinical progression (difference nonsignificant; *p* > 0.05), indicating that neuroprotective effects may depend on disease biology, dosing, and outcome measures [119]. GLP-1RAs produce measurable anti-inflammatory effects, including 20–30% reductions in IL-6 and TNF-α, and 15–25% decreases in CRP in clinical studies. Preclinical models show 30–60% reductions in ROS production, 20–40% increases in mitochondrial biogenesis markers, and 40–70% inhibition of NF-κB activation. The combination of GLP-1RA’s metabolic, anti-inflammatory (e.g., reductions in CRP and IL-6, typically *p* < 0.05), and direct neurotrophic effects in preclinical models make them among the most promising candidates for repurposing in neurodegenerative and cerebrovascular disease [97,110,120,121]. A comparative summary of cognitive outcomes for GLP-1RAs and DPP-4 inhibitors is provided in Table 2.

## 3. Therapeutic Opportunities in Neuroinflammation, Neurodegeneration, Stroke, and Cardiovascular Disorders

**Figure 6:**
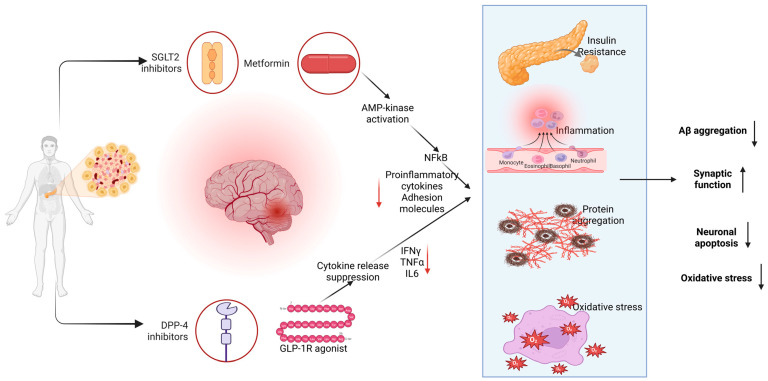
**Convergent mechanisms through which antidiabetic drug classes may modulate neuroinflammation, insulin resistance, oxidative stress, and neurodegenerative pathology.** SGLT2 inhibitors, metformin, DPP-4 inhibitors, and GLP-1 receptor agonists each exert anti-inflammatory and antioxidant effects that converge on reduced NF-κB activation, suppression of pro-inflammatory cytokines (IL-6, TNF-α, IFN-γ), and decreased expression of adhesion molecules. These systemic changes may contribute to improvements in insulin resistance, reduced oxidative stress, and attenuation of protein aggregation and neuronal apoptosis. Clinically, these drug classes have demonstrated reductions in circulating inflammatory cytokines, improvements in insulin sensitivity, and decreased systemic oxidative stress. Preclinical evidence supports additional direct neuroprotective pathways, including reductions in Aβ aggregation, improvements in synaptic function, and decreased neuronal apoptosis and oxidative stress within the CNS. The pathways shown here, therefore, represent an integration of clinically validated systemic effects with preclinical CNS-specific mechanisms, highlighting the translational potential of these therapeutic approaches.

Emerging evidence indicates that antidiabetic drugs can mitigate various neurological pathologies but also have distinct cardiovascular profiles, largely tied to their anti-inflammatory and antioxidative actions (see also Figure 6).

### 3.1. Neuroinflammation

Chronic microglial activation contributes to diseases like multiple sclerosis (MS) and traumatic brain injury. Agents that shift microglia toward an anti-inflammatory phenotype are beneficial. A strong relationship exists between peripheral insulin resistance, inflammation, and BBB dysfunction, and its role in glial activation and the exacerbation of AD pathology [122]. Metformin and GLP-1RAs both promote M2-like polarization via AMPK [123], and GLP-1RA via cAMP effects [110]. Interestingly though metformin’s effect on M2 polarization appears to be cell specific with one study indicating it suppresses M2 polarization in microglia under conditions of oxygen-glucose deprivation [124] and others showing it promotes M2 polarization in macrophages [123,125]. DPP-4 inhibitors and SGLT2 inhibitors also attenuate microglial cytokine release and support neurorepair through SDF-1α [126] and vascular modulation, respectively [127]. Although no major trials have yet tested these drugs in MS, preclinical data (e.g., metformin reducing relapses in experimental autoimmune encephalomyelitis) warrant further study [128,129].

Across neuroinflammatory conditions, AMPK, NF-κB, and Nrf2 form an interconnected signaling center rather than isolated pathways. AMPK activation in microglia and astrocytes inhibits IKK-mediated phosphorylation of IκB, thereby preventing NF-κB nuclear translocation and reducing transcription of IL-1β, TNF-α, and IL-6. At the same time, AMPK promotes Nrf2 activation (via phosphorylation and improved mitochondrial redox status), enhancing expression of antioxidant response genes such as SOD, HO-1, and catalase. In chronic neuroinflammation, failure to engage this AMPK–Nrf2 axis permits persistent NF-κB signaling, ROS accumulation, and microglial M1 polarization. By converging on AMPK activation and NF-κB inhibition while indirectly supporting Nrf2, antidiabetic agents help shift glial cells toward a less inflammatory, more reparative state, which is particularly relevant in MS, traumatic brain injury, and diabetes-associated neuroinflammation.

### 3.2. Neurodegeneration (AD/PD)

Alzheimer’s disease is marked by Aβ/tau pathology and neuroinflammation. Metformin and GLP-1RAs have both been shown to reduce Aβ accumulation and inflammatory damage in AD mouse models [130,131,132]. DPP-4is similarly attenuating AD pathology by raising protective peptides and inhibiting neuroinflammation [133,134]. In Parkinson’s disease, metformin and GLP-1RAs protect dopaminergic neurons in animal models by reducing oxidative/nitrative stress and inflammation [135,136]. Clinically, observational studies suggest that diabetic patients on GLP-1RAs or metformin have lower rates of dementia and Parkinson’s than those on insulin or sulfonylureas, but randomized trials are lacking [137].

In neurodegenerative diseases such as AD and PD, AMPK, NF-κB, and Nrf2 interact to regulate protein aggregation, synaptic integrity, and neuronal survival. Chronic NF-κB activation in glia and neurons promotes sustained release of IL-1β, TNF-α, and other mediators that exacerbate Aβ deposition, tau phosphorylation, and α-synuclein toxicity. AMPK activation can counteract this by dampening NF-κB signaling and improving mitochondrial function, thereby reducing ROS-driven damage that feeds back into inflammatory cascades. Concurrently, Nrf2 upregulation enhances antioxidant capacity and limits oxidative modification of Aβ, tau, and α-synuclein. In preclinical models, metformin, GLP-1RAs, DPP-4 inhibitors, and SGLT2 inhibitors all engage this AMPK–NF-κB–Nrf2 triad to reduce misfolded protein burden and neuronal loss. Thus, disease-specific benefits in AD and PD likely arise from coordinated modulation of these interconnected pathways rather than from single-target effects.

### 3.3. Ischemic Stroke

In animal models of stroke, all four drug classes limit brain injury and improve recovery via anti-inflammatory mechanisms. Metformin given before stroke promotes microglial M2 polarization and reduces infarct size [21,138]. GLP-1RAs acutely reduce blood–brain barrier disruption and leukocyte infiltration [103,111]. DPP-4 inhibitors (raising SDF-1α) enhance brain repair by mobilizing endothelial progenitors, while SGLT2 inhibitors (SGLT2is) improve cerebral perfusion through osmotic diuresis and neurovascular effects [139]. Clinically, GLP-1RA use in diabetics correlates with fewer strokes [140], and some retrospective studies hint that metformin improves outcomes in diabetics after stroke [47]. No large RCT has yet tested these agents in non-diabetic stroke patients for neuroprotection, but this is an active area for future research.

In ischemic stroke, the interplay between AMPK, NF-κB, and Nrf2 is particularly important during reperfusion injury. Energy failure and mitochondrial dysfunction increase ROS and activate NF-κB, which amplifies cytokine release and upregulates adhesion molecules, promoting leukocyte infiltration and BBB disruption. Timely AMPK activation can help restore metabolic homeostasis, limit excitotoxicity, and restrain NF-κB-driven transcription of pro-inflammatory genes. In parallel, Nrf2 activation enhances detoxification of ROS and supports endothelial and neuronal survival. Antidiabetic drugs that activate AMPK (e.g., metformin, GLP-1RAs) or improve mitochondrial redox balance (e.g., SGLT2i, DPP-4i) therefore intersect at this AMPK–NF-κB–Nrf2 node, collectively reducing infarct size and improving functional recovery observed in preclinical stroke models.

### 3.4. Cardiovascular Disease: Atheroprotection and Heart Failure

These antidiabetic drugs have distinct cardiovascular profiles, largely tied to their anti-inflammatory actions. In the cardiovascular system, AMPK, NF-κB, and Nrf2 similarly interact to shape endothelial function, plaque stability, and myocardial remodeling. SGLT2 inhibitors, GLP-1RAs, metformin, and DPP-4 inhibitors converge on AMPK activation and NF-κB suppression while enhancing Nrf2-mediated antioxidant defenses, thereby reducing vascular ROS, attenuating cytokine-driven fibrosis, and contributing to the reductions in heart failure events seen in clinical trials.

#### 3.4.1. Atherosclerosis

Endothelial inflammation and plaque instability underlie CAD. Metformin has shown cardioprotective effects by inhibiting pyroptosis and fibrosis, which can ultimately reduce adverse cardiovascular outcomes [141]. SGLT2is reduce arterial stiffness and oxidative stress and effectively reduce adverse cardiovascular outcomes [142,143]. DPP-4is may suppress inflammatory cytokines and MMPs, while boosting IL-10, although this is primarily supported by preclinical evidence. GLP-1RAs exert direct vascular effects: they decrease endothelial adhesion molecules, increase nitric oxide bioavailability, and reduce plaque burden in animal models. In aggregate, these drugs slow atherogenesis in preclinical studies, although only GLP-1RAs and SGLT2is have shown concrete reductions in hard events (MACE) in trials (Scheen, 2018) [144].

#### 3.4.2. Heart Failure and Myocardial Injury

SGLT2 inhibitors are particularly effective in heart failure (HF), reducing hospitalization and mortality even in non-diabetic HF patients [142,143,145]. Their diuretic effect and reduced cardiac fibrosis and inflammation underpins this benefit. GLP-1RAs also improve cardiac function; liraglutide reduces infarct size after MI in animals by limiting inflammatory cell infiltration. DPP-4is modestly increasesGLP-1/SDF-1 levels to help myocardial repair, although one trial noted an increase in HF hospitalization with saxagliptin (a finding not seen with other DPP4i). Metformin has shown reduced cardiac fibrosis in animal models and improved outcomes in observational HF cohorts [141,146].

#### 3.4.3. Clinical Trials

Multiple large trials confirm these benefits: EMPA-REG OUTCOME, CANVAS, and others showed SGLT2is cut CV events and HF hospitalizations dramatically; GLP-1RA trials (LEADER, SUSTAIN-6, etc.) showed 10–14% reductions in MACE and significant stroke reductions [144]. Conversely, DPP-4 inhibitor trials were neutral on MACE [147]. These findings underscore that SGLT2is and GLP-1RAs confer broad cardio-protection likely via combined metabolic and anti-inflammatory pathways [148].

## 4. Future Directions and Emerging Therapies

Given these promising effects, future research is expanding in several directions. One focus is novel incretin-based therapies: dual or triple agonists (e.g., GLP-1/GIP analogs like tirzepatide, or GLP-1/GIP/glucagon agonists) may combine and amplify the benefits regarding inflammation, metabolism, and neural repair [149,150]. Highly brain-penetrant analogs or conjugates (e.g., modified GLP-1 analogs that cross the blood–brain barrier more effectively) are under investigation, as preliminary data suggest agents like dulaglutide have excellent CNS uptake [151]. Clinical trials of GLP-1RAs in primary neurodegenerative disease cohorts are underway (e.g., semaglutide in Alzheimer’s disease) [152], while dedicated stroke recovery trials could test these agents as adjunctive therapy. Biomarker studies (e.g., imaging of microglial activation, cytokine profiling) will help clarify which patient subsets benefit most. Across drug classes, treatment consistently results in 10–30% reductions in pro-inflammatory cytokines (IL-6, TNF-α, CRP) and 30–50% decreases in ROS levels, with corresponding increases in antioxidant enzyme activity.

There is also interest in repurposing these drugs in non-diabetic individuals at high risk of neuro-cardiovascular disease. For example, metformin is being tested for cognitive decline in aging-related trials [153], and SGLT2is are being studied in vascular dementia models [154,155]. Finally, combination strategies (e.g., adding a GLP-1RA to standard anti-inflammatory treatment in MS or dementia) or new delivery systems (targeting drugs directly to microglia or endothelium) may emerge. The intersection of metabolism, inflammation, and neurovascular biology is a rich field; harnessing the anti-inflammatory and antioxidant properties of antidiabetic agents holds significant promise for future therapies (see Table 3).

Future clinical trials should prioritize patient populations at high risk of cognitive decline or cerebrovascular injury-such as older adults with T2D, metabolic syndrome, microvascular disease, or prior stroke and incorporate mechanistic biomarkers that reflect inflammation, oxidative stress, and neurodegeneration. Trials testing SGLT2 inhibitors, GLP-1 receptor agonists, metformin, or DPP-4 inhibitors should include plasma and CSF biomarkers such as IL-6, TNF-α, CRP, MMP-9, NfL, GFAP, Aβ42/40 ratios, p-tau species, oxidative stress markers (e.g., 8-oxo-dG, MDA/TBARS), and mitochondrial function readouts. Neuroprotective outcomes should be assessed with FDG-PET cerebral metabolism, MRI markers of microvascular injury (white matter hyperintensities, hippocampal volume), and sensitive cognitive composites to detect early changes in memory, executive function, and processing speed.

Given the strong cardiometabolic effects of these drug classes, future trials should also integrate cardiovascular endpoints such as heart failure hospitalization, major adverse cardiovascular events (MACE), arterial stiffness, endothelial function (flow-mediated dilation), and biomarkers of vascular inflammation. Pragmatic or hybrid RCT designs comparing GLP-1RAs or SGLT2 inhibitors against standard care, with long-term follow-up for dementia and stroke incidence, would help determine true disease-modifying potential. Ultimately, combining mechanistic biomarkers with clinically relevant neurological and cardiovascular endpoints will clarify which antidiabetic agents are most likely to provide durable neuroprotection.

## 5. Implications for Longevity and Healthy Aging

Growing interest in the intersection between metabolism, inflammation, oxidative stress, and aging has positioned several antidiabetic agents—particularly metformin, SGLT2 inhibitors, and GLP-1 receptor agonists—as potential geroprotective therapies. Aging is characterized by progressive mitochondrial dysfunction, chronic low-grade inflammation (“inflammaging”), impaired stress-response signaling, vascular stiffness, and metabolic inflexibility. These processes converge mechanistically with the pathways implicated in neurological and cardiovascular disorders, including AMPK activation, NF-κB suppression, Nrf2-mediated antioxidant defense, impaired autophagy, and glial dysregulation. As a result, pharmacologic agents that modulate these pathways in diabetes and cardiometabolic disease may also ameliorate fundamental biological processes that drive aging and age-related decline.

**Metformin** is the most established example: through AMPK activation, improved mitochondrial function, and suppression of inflammatory signaling, metformin has been shown in preclinical studies to extend lifespan in multiple model organisms and improve health span metrics such as mobility, cognition, and vascular function. Its broad effects on oxidative stress, endothelial repair, and neuroinflammation have fueled major translational efforts including the Targeting Aging with Metformin (TAME) initiative to evaluate whether metformin can delay age-associated morbidities even in non-diabetic populations. The mechanistic overlap highlighted in this review (e.g., AMPK–NF-κB–Nrf2 crosstalk, mitochondrial homeostasis, modulation of neuroinflammation) aligns closely with pathways thought to underlie healthy aging, making metformin a leading candidate for geroscience-directed interventions.

**GLP-1 receptor agonists** have recently entered longevity discussions due to their ability to reduce chronic inflammation, improve metabolic flexibility, enhance mitochondrial quality control, and modulate neuroimmune signaling—all processes that deteriorate with age. Early imaging studies suggesting stabilization of brain glucose metabolism, alongside their well-documented cardiometabolic benefits and anti-inflammatory properties, support the hypothesis that GLP-1RAs may slow certain facets of biological aging, particularly in the CNS and vascular system. Their capacity to target multiple organ systems involved in aging (brain, vasculature, adipose tissue, and immune compartments) further strengthens their potential relevance to health span.

**SGLT2 inhibitors** also modulate biological pathways increasingly linked to longevity. By attenuating systemic inflammation, lowering oxidative stress, promoting ketone metabolism, improving mitochondrial efficiency, and reducing cardiac and renal workload, SGLT2 inhibitors reproduce metabolic states associated with lifespan extension in preclinical models (e.g., reduced insulin signaling, enhanced fatty acid oxidation, increased autophagy). Their benefits in non-diabetic heart failure populations further suggest utility beyond glycemic control, including possible relevance to age-associated cardiovascular and neurovascular dysfunction.

**DPP-4 inhibitors**, though less frequently discussed in geroscience, influence oxidative stress pathways, immune aging, and neuroinflammation through increased endogenous incretins and modulation of SDF-1α. While current evidence is more limited, their capacity to reduce inflammatory cytokines and improve antioxidant defenses points to potential roles in mitigating immune-senescence and age-related vascular decline.

Collectively, these mechanistic and translational observations underscore that antidiabetic agents not only treat metabolic disease but also target biological hallmarks of aging, including mitochondrial dysfunction, chronic inflammation, proteostasis imbalance, impaired stress resistance, and vascular deterioration. Their relevance to longevity medicine therefore provides additional justification for evaluating these agents across neurological and cardiovascular conditions. Future aging-focused clinical trials—ideally incorporating biomarkers of inflammation, oxidative stress, biological age, and neurovascular function will be essential to determine whether these therapies meaningfully extend health span or delay the onset of age-related neurological and cardiovascular diseases.

## 6. Conclusions

Antidiabetic agents exert important anti-inflammatory, antioxidant, and neuroprotective effects that extend beyond glucose-lowering effects, but their clinical relevance varies across drug classes. Among currently available therapies, SGLT2 inhibitors and GLP-1 receptor agonists show the strongest clinical promise based on consistent reductions in cardiovascular events, improvements in systemic inflammatory markers, and emerging signals for cognitive protection. SGLT2 inhibitors demonstrate robust benefits for heart failure and cardiovascular mortality across large CVOTs, with parallel reductions in IL-6, TNF-α, and oxidative stress that may translate to stroke and dementia risk reduction. GLP-1RAs combine metabolic improvements with favorable effects on inflammation, endothelial function, and mitochondrial health; early Alzheimer’s trials and large observational datasets suggest potential disease-modifying effects that merit further testing. Metformin, although supported by observational dementia studies and strong preclinical neuroprotective data, requires modern randomized trials to confirm cognitive or cerebrovascular benefit. DPP-4 inhibitors remain cardiovascularly safe and show intriguing epidemiological associations with reduced dementia and Parkinson’s risk, but dedicated neurological outcome studies are lacking.

Together, these findings highlight that antidiabetic drugs modulate interconnected pathways—AMPK, NF-κB, Nrf2, mitochondrial redox balance, and glial activation—that are central to neurodegeneration and cerebrovascular dysfunction. The practical implication is that future clinical trials should incorporate mechanistic biomarkers (e.g., neuroinflammatory cytokines, oxidative stress markers, FDG-PET, plasma NfL, cognitive composites) to clarify which pathways most strongly predict therapeutic response. Designing disease-specific, adequately powered RCTs in populations at high risk of dementia or stroke (older adults with T2D, metabolic syndrome, or microvascular disease) will be essential to translate promising preclinical results into clinically actionable therapies. In summary, while several antidiabetic drug classes possess neuroprotective potential, current evidence most strongly supports SGLT2 inhibitors and GLP-1RAs as leading candidates for repurposing in neurodegenerative and cerebrovascular disease. Continued integration of mechanistic insights with rigorous clinical testing will be critical for defining their role in neurological prevention and treatment.

## Figures and Tables

**Table 1:** **Summary of Clinical and Observational Evidence on DPP-4 Inhibitors in Cardiovascular and Neurological Outcomes.** This table synthesizes findings from major cardiovascular outcome trials (CVOTs) and observational studies on DPP-4 inhibitors. It highlights their cardiovascular safety (MACE neutrality), potential neuroprotective effects (dementia and Parkinson’s risk), and the current gap in large-scale neurological trials confirming preclinical anti-inflammatory benefits.

Trial/Study	Focus	Finding
EXAMINE	CVOT (Alogliptin)	MACE-neutral
SAVOR-TIMI 53	CVOT (Saxagliptin)	MACE-neutral; ↑ HF risk
TECOS	CVOT (Sitagliptin)	CV-safe
CAROLINA	Linagliptin vs. Glimepiride	Non-inferior on MACE
CARMELINA	CV/Kidney (Linagliptin)	No ↑ risk; renal safety
Kim et al. (2019) [89]	Dementia incidence	↓ Risk in DPP-4i users
Maanvi et al. (2022) [90]	Parkinson & cognition	Reviews ↓ incidence data
Calabrese et al. (2021) [91]	Aging, T2DM, neuro	Metformin + DPP-4i ↓ Parkinson risk
Epelde (2024) [92]	CV & neuro review	Need for neuro RCTs

↑ = Increase; ↓ = Decrease.

**Table 2:** **Comparative Cognitive Outcomes for GLP-1RAs vs. DPP-4 Inhibitors.** Summary of comparative cognitive and neuroprotective outcomes observed with GLP-1 receptor agonists and DPP-4 inhibitors. Values include effect sizes (HRs, ORs, and mean differences) and corresponding statistical significance from randomized trials and large observational cohorts.

Drug Class	Study	Sample Size	Cognitive/Dementia Outcome	Effect Size (HR, OR, Score)	Statistical Significance
GLP-1RA (liraglutide)	Mild Alzheimer’s RCT (6months)	N = 38	Stabilized brain glucose metabolism (FDG-PET) vs. placebo; no difference in cognitive test scores	Δ FDG-PET decline: −0.024 vs. −0.039 SUVR	*p* ≈ 0.01 for PET; NS for cognition
GLP-1RA (dulaglutide)	REWIND post hoc cognitive analysis	N = 8828	Slower cognitive decline on composite cognitive score compared to placebo	Mean difference: −0.058	*p* = 0.026
GLP-1RA	Observational dementia cohorts (older adults with T2D)	N = 50,000–200,000	Lower incidence of dementia compared with non-GLP-1 users	HR 0.75–0.82	*p* < 0.05
DPP-4 inhibitors (class)	Observational dementia cohort (T2D + metformin)	N ≈ 30,000	Lower risk of dementia vs. other regimens; slower cognitive decline	HR 0.75–0.85	*p* < 0.05
DPP-4 inhibitors (class)	Parkinson’s risk cohort	N ≈ 20,000	Reduced incidence of Parkinson’s disease	~15–20% risk reduction	*p* < 0.05
DPP-4 inhibitors (EXAMINE, SAVOR, TECOS)	CVOTs with cognitive outcomes not measured	N = 5000–16,500	No cognitive endpoints measured	-	-

**Table 3:** Neuroprotective Potential and Limitations of Major Antidiabetic Drug Classes.

Drug Clas	Key Neuroprotective Mechanism	Observed/Proposed Benefits	Major Limitations & Challenges	Translational Opportunities
**Metformin**	Activates AMPK, suppresses mTOR, enhances mitochondrial biogenesis, reduces oxidative stress and inflammation	↓ Aβ and tau phosphorylation; ↑ autophagy; improved neuronal metabolism and cognitive performance (in preclinical studies)	Limited BBB penetration; inconsistent clinical outcomes; risk of lactic acidosis in renal impairment; unclear long-term safety in non-diabetics	Develop brain-penetrant analogs; combine with lifestyle/metabolic interventions; biomarker-guided prevention trials
**GLP-1R Agonists**	Activates GLP-1R → cAMP/PKA pathway; anti-inflammatory; anti-apoptotic; enhances synaptic plasticity	↓ Microglial activation, ↓ Aβ, ↓ tau pathology, ↑ cognition; dual benefit for cardiovascular health	Injectable route; GI side effects; high cost; uncertain CNS exposure in humans	Optimize CNS delivery (intranasal/analog design); evaluate long-term neurovascular effects in ongoing AD trials (EVOKE/EVOKE+)
**SGLT2 Inhibitors**	↓ Glucose toxicity, ↓ oxidative stress, improves mitochondrial function and BBB integrity	Improved cognitive outcomes in diabetic patients; ↑ cerebral perfusion; ↓ inflammation	Limited mechanistic CNS data; dehydration and ketoacidosis risk; low BBB penetration	Conduct dedicated cognitive trials; study neurovascular coupling and ketone metabolism; explore brain-targeted analogs
**DPP-4 Inhibitors**	↑ Endogenous GLP-1, ↓ cytokines (TNF-α, IL-6), ↑ Nrf2-mediated antioxidant response	↓ Neuroinflammation and oxidative stress; potential neurovascular protection	Modest CNS penetration; indirect mechanism may limit efficacy; minimal cognitive clinical data	Combine with GLP-1R agonists for additive benefit; biomarker validation for inflammation/oxidative stress modulation
**TZDs (PPARy agonists)**	Activates PPARγ → inhibits NF-κB, reduces oxidative stress and glial activation	↓ Neuroinflammation; improved mitochondrial function and vascular health	Mixed clinical trial outcomes; weight gain, edema, cardiac risk	Develop selective PPARγ modulators; stratify trials by insulin resistance and inflammatory biomarkers

↓ = Decrease; ↑ = Increase; → = Effect

## Data Availability

No new data were created or analyzed in this study.

## Abbreviations

AMPK: AMP-activated protein kinase; Nrf2, nuclear factor erythroid 2-related factor 2; NF-κB, nuclear factor kappa B; NLRP3, NLR family pyrin domain containing 3 inflammasome; GLP-1R, glucagon-like peptide-1 receptor; PI3K, phosphoinositide 3-kinase; Akt, protein kinase B; ROS, reactive oxygen species; BDNF, brain-derived neurotrophic factor; BBB, blood–brain barrier; MACE, major adverse cardiovascular events; HF, heart failure; AD, Alzheimer’s disease; PD, Parkinson’s disease; SGLT2i, sodium-glucose cotransporter-2 inhibitors; DPP-4i, dipeptidyl peptidase-4 inhibitors; GLP-1RA, GLP-1 receptor agonist; PP2A, Protein Phosphatase 2A.
